# Environmental Distribution of Personal Care Products and Their Effects on Human Health

**DOI:** 10.22037/ijpr.2021.114891.15088

**Published:** 2021

**Authors:** Madiha Khalid, Mohammad Abdollahi

**Affiliations:** a *Toxicology and Diseases Group, Pharmaceutical Sciences Research Center (PSRC), The Institute of Pharmaceutical Sciences (TIPS), Tehran University of Medical Sciences (TUMS), Tehran, Iran. *; b *Department of Toxicology and Pharmacology, School of Pharmacy, Tehran University of Medical Sciences, Tehran, Iran. *

**Keywords:** Environment, Health, Personal care products, Phthalates, Toxicology, Ultraviolet filters

## Abstract

Personal care products (PCPs) are generally used for personal hygiene, cleaning, grooming, and beautification. These include hair and skin care products, baby care products, UV blocking creams, facial cleansers, insect repellents, perfumes, fragrances, soap, detergents, shampoos, conditioners, toothpaste, *etc.*, thus exposing humans easily. Personal preferences related to PCPs usage frequency are highly variable and depend on socioeconomic status and lifestyle factors. The increasing availability and diversity of PCPs from the retailer outlets consequently result in higher loading of PCPs into wastewater systems and, therefore, the environment. These compounds persistently and continuously release biologically active and inactive ingredients in the atmosphere, biosphere, geosphere, and demonstrating adverse effects on human, wild, and marine life. Advanced techniques such as granular activated carbon filtration and algae-based system may help biotransformation and remove PCP contaminants from water with improved efficiency. Additionally, harmony among PCPs related regulations of different countries may encourage standard checks to control their manufacturing, sale, and distribution across the borders to ensure consumers’ safety. Furthermore, all intended ingredients, their concentrations, and instructions for frequency of use as per age groups may be clearly labeled on packages of PCPs. In conclusion, the emerging environmental contaminants of PCPs and their association with the growing risks of negative effects on human health and globally on the environment emphasize the chemical-free simple lifestyle.

## Introduction

Personal care product is a category of self-care products generally used for personal hygiene, cleaning, and grooming. There are several sources for the environmental emerging contaminants, whereas PCPs are one of them (1). These are the highly prevalent category of consumer products at home and in public places. Nearly 30-40% of dermatologist’s prescriptions worldwide contain at least one item of PCPs, and an individual uses at least two PCPs for 24 h (2, 3). In the Hunan Province of Southern China, the total mass loading of PCPs was 506.35 mg/d/1000 people contributing to the total emission of 357.56 mg/d/1000 people (4). PCPs are the source of emission of several chemicals. For example, only shampoo and shower gel are sources of per capita siloxanes emission of 49.25 and 9574 μg/day. Similarly, decamethylcyclopentasiloxane (D5) and dodecamethylcyclohexasiloxane (D6) were found prevalent in the air with per capita emission values of 8.33 and 6109 μg/day (5). Likewise, phthalates from PCPs contaminate indoor air (6). Monoethanolamine, and diethanolamine commonly found in cleaners, shampoos, hair dyes, and detergents (7). TESIE study identified phthalates in almost all hand wipes and dust samples and their metabolites detected in all children’s urine samples, confirming its ubiquitous exposure (8). 

While commonly used, these products can contain chemicals undisclosed to the public through labels. Nearly 80% of college women did not know if their PCPs were free of endocrine-disrupting compounds (EDCs), *e.g.*, phthalates (3). Likewise, 48.6% of female college students were uncertain about the safety of PCP use. Moreover, 79% of female college students were concerned about the impact of PCPs contents on their reproductive health (9). Human is exposed to PCPs throughout their lifetime, even during intrauterine development. The direct routes of exposure are inhalation, dermal contact, ingestion, and absorption, while indirect ways involve using other products and environmental contamination (10). Inhalation exposure will be relatively low when a product is used in a ventilated area (11). For example, the estimated daily dermal route of exposure of titanium dioxide (TiO_2_) ranges from 2.8 to 21.4 mg/person/day, and sunscreen and toothpaste are significant contributors to TiO_2_ dermal exposure.

Moreover, 0.15 to 3.9 mg TiO_2_/day were estimated to be ingested through the toothpaste. It is estimated that nearly 35% of manufactured TiO_2_ is used in PCPs as UV protecting agents or to prevent product discoloration (12). Effects of PCPs often last for weeks by producing an individualized response through altered steroid/pheromone levels and changed bacterial/archaeal diversity (13). Chemicals in PCPs have a high health risk to human and aquatic life (14). Little information is available about exposures from PCPs and some ingredients of PCPs known as EDCs and involved in abnormal developmental and reproductive ability. Consumer knowledge about the emerging contaminant of PCPs in humans is essential to raise awareness regarding their toxicity towards human beings. Metabolism, biotransformation, and later bioaccumulation increased severe health issues related to EDCs. Hereby we have described different aspects of the emerging contaminant of PCPs, their sources, and routes of exposure, environmental distribution, the pattern of consumer usage, and related health risks. Finally, we concluded some new recommendations that meeting them may reduce environmental exposure to emerging PCPs. 

Definition and classification

Various products have been used for health and beauty that are generally termed ‘personal care products.’ Such products include skin moisturizers, perfumes, lipsticks, fingernail polishes, eye and facial makeup preparations, shampoos, hair colors, toothpaste, deodorants, *etc.* At the same time, some may contain drugs such as protectants, lip balms, diaper ointments, mouthwashes, and antiperspirants. PCPs include both categories of cosmetics and medicines that are utilized for aesthetic and therapeutic purposes. Therefore, they meet the definition of cosmetics and/or drugs, *e.g.*, toothpaste for cleaning and their fluoride content for strengthening teeth and moisturizers for soothing skin. Their UV-filters protect against harmful sun rays. In Canada, PCPs defined as a substance or mixture of substances that are generally recognized by the public for use in daily cleansing or grooming, therefore, grouped into three regulatory categories, namely cosmetics, drugs, or natural health products (1, 15). The term ‘personal care product,’ however, is not yet defined by US FDA law. Therefore, in the US, commonly referred to as ‘personal care products’ regulated as either cosmetics or drugs or both if a product has two intended uses, *e.g.*, medicated antidandruff shampoo used to clean hair and to treat dandruff. Hence a PCP can be defined as a nonprescription self-care product used for personal hygiene, cleaning, grooming, and beautification. The emerging contaminants of PCPs are grouped into 1. alkylphenol polyethoxylates (APEOs), 2. antimicrobials, 3. bisphenols, 4. cyclosiloxanes, 5. ethanolamines, 6. fragrances, 7. glycol ethers, 8. insect repellents, 9. parabens, 10. phthalates and 11. UV filters. 

Regulations

PCPs to date are not adequately defined by any national or international laws. This class of products has been widely used and made available to the public without proper regulations. Some PCPs containing drugs like antibacterial soaps, antiperspirant deodorants, sunscreens, *etc.*, are regulated as over-the-counter products according to US-FDA, though they require the listing of ‘active ingredients’ on the label (16). Missing ingredients information on the PCPs label causes many problems from multiple perspectives. First of all, the consumer fails to get exact information about the contents of the PCPs and cannot make any precautionary selection of a product for personal use. Secondly, researchers fail to get authentic ingredients information of PCPs for research-related test analysis, test exposure, or epidemiological surveys.

Additionally, manufacturers are somewhat reluctant to provide exact information about the products’ contents due to the gaps in different legislation (7). More than 1500 chemicals in Europe and 800 chemicals in Canada are banned or restricted from PCPs, while interestingly, only 11 substances are prohibited or restricted in the US (17). Nearly 45% of soaps in the US reported containing antibacterial agents, especially TCS, despite limiting their US-FDA usage (18, 19). Limitations within the rules and regulations result in unnecessary chemical exposure and uncontrolled distribution of different drugs containing PCPs. For example, US law does not require disclosing all chemical ingredients, *e.g.*, fragrances (20).

Furthermore, the US manufacturers do not require to mention the safety or efficacy of the cosmeceuticals before marketing (2). Such unclear rules and regulations left loopholes for ambiguous and inadequate product handling by different regulatory authorities. For example, the US regulates antidandruff shampoos, antiperspirants, and sunscreen as a drug while they are classified as cosmetics in Europe. Cosmeceuticals are a subclass of cosmetics in Europe and Japan, while a subclass of medicines in the US (2). The absence of specific PCPs related rules and lack of harmony among different countries’ existing regulations fail to ensure humans’ safety due to their uncontrolled exposure through air, water, soil contaminations of PCPs, and their wastes.

Additionally, the lack of sufficient evidence-based information and databases further halt regulatory authorities to regulate pharmaceuticals or manufacturers regarding controlled and safe chemical use in the PCPs. For example, the FDA failed to conclude any health hazards with phthalate esters’ exposure through cosmetic use due to insufficient data (21). However, literature identified that PCPs may contain more than hundreds of volatile organic compounds (VOCs) and reported the presence of nearly 24 chemicals in sunscreen (7, 20). Some of them are regulated as toxic or hazardous chemicals and classified as ‘Hazardous Air Pollutants’, but unfortunately, none of such substances were listed on any product label (20). It is also found that many chemical compounds even persist after recycling (8). No PCPs label listed chemicals such as monoethanolamine, diethanolamine, fragrances, alkylphenols, or surfactants, *etc.*, as an ingredient. Thus the consumer is, in fact, unable to avoid exposure to such chemical through mere reading the product labels (7). With increasing global concern, the US Consumer Product Safety Commission imposed a ban on phthalates including BBP, di-n-butyl phthalate (DBP), di-isobutyl phthalate (DiBP), di (2-ethylhexyl) phthalate (DEHP), and di-isononyl phthalate (DiNP) in various products.

Similarly, European Chemical Agency banned BBP, DBP, DEHP, and DiBP in all European Union products. Despite bans, manufacturers found to replace phthalate esters with several plasticizers such as di (2-ethylhexyl) terephthalate and diethylhydroxylamine (8). The facts mentioned above highlight the need for common grounds and criteria for defining PCPs as a separate class of products independent of cosmeceuticals, drugs, and other regulated products. There is a need to establish a mechanism to harmonize the rules and regulations of different authorities to have standard checks and control for their manufacturing, sale, and distribution across the borders to ensure consumer’s safety and efficacy. 

Sources and their occurrence

PCPs are one of the sources of environmental contaminants through human usage and thus ubiquitous. Alkylphenol polyethoxylates such as nonylphenol and octylphenol ethoxylates are commonly used as surfactants in PCPs, while alkylphenol is the breakdown product of APEOs. APEOs are found in detergents, disinfectants, surface cleaners, and different PCPs (7). Some PCPs also contains antimicrobials due to their aseptic property. The most commonly used antimicrobial agents of PCPs are ortho-phenylphenol, triclocarban, triclosan, and 1,4-dichlorobenzene. Such antimicrobials are commonly found in toothpaste, soaps, deodorants, and detergents (7, 22). Bisphenols are used in plastic productions, *e.g.*, epoxy resins and polycarbonates. These are not the intended ingredient of PCPs and merely present due to their migration from plastic containers or degradation (23). The most common Bisphenol A of PCPs identified in detergents, soaps, lotions, shampoos, conditioners, shaving creams, nail polishes, and sunscreens (7). Cyclic volatile methyl siloxanes or cyclosiloxanes and linear siloxanes have spreading and conditioning properties thus used in PCPs such as baby products, sunscreen, shaving creams, cleaners, hair-care products, lotion, body washes, and cosmetics (7, 24). Low molecular weight cyclosiloxanes were detected in nearly 252 different PCPs in Canada (25). Ethanolamines are ammonia compounds that act as a surfactant and have antistatic, conditioning, emulsifying, foaming, and viscosity increasing property thus used in PCPs and cosmetics (26). Fragrances and perfumes contain various chemicals. There are 50-300 different chemicals used as fragrances for PCPs. Such chemicals may be acetals, alcohols, aldehydes, amides, amines, carboxylic acids, coumarins, dioxanes, epoxides, esters, ethers, heterocyclics, hydrocarbons, ketones, lactones, musks, nitriles, phenols, pyrans, pyrazines, quinolines, or Schiff’s bases (27). Several PCPs such as detergents, soaps, cleaners, and fabric softeners contain various fragrances (27). PCPs, including perfumes, lotions, deodorants, body cream, facial cleanser, and sunscreen, usually have a higher concentration of perfumes or even synthetic fragrances (7, 28). Glycol ethers, due to their hydrophilic and lipophilic nature used as a cleaning agent in cleaners, face lotion, shaving creams, and sunscreen (7). The US introduced N, N-diethyl-m-toluamide or DEET as an insect repellent against mosquito bites in 1946 for their army. The Environmental Protection Agency and the Centers for Disease Control and Prevention recommended that DEET is the first-line mosquito repellent (29). Bayrepel, indole, and piperonyl butoxide are other commonly used chemicals as insect repellent (30). Parabens are used as preservatives in PCPs (31). Phthalates or phthalic acid esters have been widely used in PCPs as additives and plasticizers. The most commonly used phthalates are diethyl phthalate, dimethyl phthalate, di-isobutyl phthalate, di-n-butyl phthalate, and di(2-ethylhexyl) phthalate. These are commonly found in cosmetics, fragrances, hair products, baby products, skin cleansers, and nail polishes (32). There observed increasing trends of phthalates replacement by di(2-ethylhexyl) terephthalate (33). PCPs for dermal applications often contain a UV blocking agent or UV filters. Skin lotions, makeup and sunscreens reported a higher concentration of benzophenone-3 (BP3) as a UV blocking agent (34). Thus a human is exposed to a variety of chemicals through a mere small package of PCP. Common sources of environmental contaminants in PCPs are presented in Table 1. 

The usage pattern of personal care products 

Few datasets are available on the PCPs usage pattern in different populations. Today’s men are prone to exposure to multiple PCPs at a time and thus potentially exposed to a more extensive mixture of compounds each day. Excessive use of PCPs highlight the importance of considering the cumulative toxicological effects of combined exposure to multiple chemicals (7). Conducting epidemiological studies is crucial for gathering information required for safety assessments, consumer exposure, or toxicological risk assessment. Such tasks require the information on contents and ingredients of PCPs, their amounts, and frequency of use among different age groups (42-44). Besides, it is essential to identify simultaneous exposure to a mixture of compounds and chemicals from all possible sources and routes of PCPs exposures to conduct aggregate exposure assessments. PCPs exposure levels vary significantly among consumers and various products due to their different behavior patterns (45).

Furthermore, the type of ingredients of PCPs, the extent of PCPs usage, dermal penetration, metabolism, co-use, and non-use patterns of PCPs affect the overall estimation of aggregate exposure to ingredients (46). The personal preferences related to PCPs usage frequency are highly variable and depend on socioeconomic status and lifestyle factors. Factors such as age, climate, education level, gender, race, and survey season considerably affect the variability of PCPs use patterns (47). The amount of PCPs usage and application area also differs from the product type, season, and country. In addition to the factors mentioned above, occupation and income are also found to significantly alter the usage pattern of various PCPs (1, 48). 

American women are regular users of several PCPs such as face cream, body lotion, and lipstick. The adverse impact of several applications used daily depends on the body area. Products such as body wash, eye shadow, facial cleanser, liquid foundation, perfume, and shampoo are other commonly used PCPs by females (42, 43 and 49). Likewise, nearly 30 PCPs are regularly utilized in California households and their frequency of use found to vary by gender and consumer’s age (47). The reproductive age of females was also found to influence the PCPs usage patterns. For example, the use of cosmetics and hair styling products declined during pregnancy or the postpartum period. Simultaneously, the use of baby products such as baby wash, baby lotion, diaper cream, *etc.*, increases after the baby’s birth (1).

Moreover, females were found to use lipstick most often while perfume among males (48). Likewise, women are more likely to use PCPs than men and like to use certain PCPs more often than men. However, a similar number of both males and females found using liquid soaps and special care products (47) except shaving products (11). Like adults, the usage pattern of PCPs also varies among children. The younger children commonly use bath gel and body lotions, while older children use antibacterial soap, body lotion, hair mousse, hair conditioner, lip balm, and nail polish. Children of more than eight years of age are more likely to use hair conditioners and deodorants. However, girls were found to use more PCPs than boys though less frequent sex differences were found among children as far as the type of PCPs usage is concerned (47). Race and ethnicity also affect the prevalence and frequency of PCPs use patterns. For example, African Americans have different hair textures. They treat them permanently with chemical straightening and relaxing agents, thus consuming shampoo and hair conditioner less frequently than Asian females.

In contrast, skincare products are more prevalent among Asian females (47). There also observed age-based variability among PCPs usage patterns. Young females were found to use more PCPs than older females. Young girls mostly use hair mousse, shampoo, and conditioner. In contrast, products related to personal grooming such as hair dye, nail polishes, mascara, foundation, hair spray, *etc.* found more prevalent among older females (11, 47). Younger girls of less than ten years were found to use sun spray and sun cream in South Korea than older girls (50).

Similarly, the annual use of hair dye among women varies with the product type and age (51). Among females, the use of chemical relaxers or straighteners was found to increase from childhood to adolescence while decreases in adulthood (52). Comparatively, younger males like to use hair mousse and sunscreen more often than older males who commonly use aftershave products and hair sprays (11, 47). 

The usage pattern of certain PCPs also associated with the different seasons, *e.g.*, lip balm was highly prevalent among consumers during the dry summer season in California (47). The PCPs type and gender were also found to influence the amount of product use. Generally, skincare products such as body lotion, creams, or sunscreen are consumed in large quantities. Women use more amount of skincare, after sun care, and tanning products than men. Similarly, women consume more shaving products than men, probably due to their comparatively larger shaving areas. Age was also found to affect the amount of PCPs usage; for example, a higher amount of eye shadow was prevalent among younger and middle-aged women than older-aged women (11). The exposure levels of different environmental contaminants also vary with the application area of PCPs. Since the total exposure to PCPs varies with skin permeability of anatomical sites. The skin permeability decreases in the order of scrotum < forehead < axilla < scalp < back < extremities. Some products such as body lotion, sunscreen, and skincare products are not restricted to a specific body part and thus relatively applied to large areas in a larger amount. Nearly 90% of consumers use body lotions on legs, upper and lower arms. Men apply shaving foam on the head and face while women on their axillae, pubic area, and lower legs. Shaving foams was found more prevalent among young consumers than middle and senior consumers (11). Some product types are also used at different times of the day, such as bathing foam, night creams, make-up or nail polish remover, *etc.*, mostly consumed during the evening or night. Some products are consumed during any time of day, such as a hand cream and lip balm (11).

The education level of a consumer was also found to influence the usage pattern of PCPs. People with a high level of education were found to use aftershave, hair dye, and apply more eye pencils than people with an intermediate or low level of education (11, 47). Nevertheless, more than 70% of consumers found to use scented than unscented PCPs, possibly due to the unavailability of unscented products in the market. However, the utilization of unscented hygiene products was higher among children than adults (47). Biesterbos and colleagues also investigated the co-use pattern of 32 different PCPs. On average, women use 17, while men use seven different PCPs with an overall of 13 PCPs by both genders. On an age basis, on average, 11, 14, and 16 PCPs products were used by senior, middle-aged and young consumers, respectively (11). 

Distribution routes of PCPs in environmental compartments

The increasing availability and diversity of PCPs from the retailer outlets consequently result in higher loading of PCPs into wastewater systems and, therefore, the environment (30). Thus the biologically active and inactive ingredients of PCPs are persistently and continuously releasing into the atmosphere (53). The primary exposure route of PCPs is the municipal wastewater. During their regular activities such as bathing, cleaning, showering, and washing, households and consumers dispose of PCPs in toilets. The non-biodegradable PCPs discharges through the wastewater treatment plants enter the receiving waters (30, 54). Therefore, the efficiency of wastewater treatment plants (WWTPs) is crucial for the removal of PCPs. Moreover, the lipophilic ingredients of PCPs sorb onto the sludge and sediments. Such digested sludge can contaminate agricultural land, soil, and runoff when used as fertilizers. Wastes from the manufacturing plants directly pollute the local environment. Landfill leaks contaminate groundwater, and odors contaminate surrounding air. Other possible exposure routes of PCPs are leakages from septic tanks, manure storage tanks, and sewer discharges (54). Such environmental contaminants of PCPs reach all different environmental compartments, including raw wastewater, wastewater effluent, surface water, groundwater, drinking water, wastewater solids, and sediment (30). Several factors influence the environmental distribution of PCPs contaminants, such as temporal changes during the dry and wet season, physical-chemical properties of PCPs ingredients, dilution factor, concentration, organic carbon content, and mode of action (55, 56). 

 Despite the ban on APEOs and alkylphenols in Europe, they are still found in surface waters of Portuguese and wastewaters of Western Balkan, including Bosnia and Herzegovina, Croatia, and Serbia. This might be due to the poor wastewater management practices causing the contaminants from wastewaters to reach ambient water (57, 58). Higher concentrations of nonylphenol and octylphenol were identified, *i.e*., 26.5 ng/L and 113 ng/L in Sail Rock Beach and Boli River estuary of Taiwan, respectively (35). Alkylphenols were also detected in California’s indoor and outdoor air, suggesting frequent prevalence in the environment (59). Downstream water samples of WWTPs in Pecan Creek, Denton, Texas, reported antimicrobials including TCS and TCC in the concentrations between 50-200 ng/L (60). TCC was detected in a range of 2-84 ng/g in India’s Hooghly river (61). Such biocides and their metabolites are lipophilic and generally not entirely removed by the WWTPs and thus detected in the direct discharge of wastewater and wastewater effluent (55, 60, 62). Moreover, the pH of an environment changes the partition coefficient (log D) of TCS. The log D values of 4.9, 4.8, and 3.7 of TCS were observed at pH 6, 7.5, and 9, respectively, suggesting that the fate and distribution of biocide are highly dependent on water quality (30). TCS and its metabolite methyl-triclosan were also detected in Switzerland’s lakes and rivers in 74 and 2 ng/L concentrations, respectively. Greifensee lake of Switzerland indicated significant decomposition and removal of a dissociated form of TCS during summer than winter season when the half-life of TCS decreases to less than an hour. However, nondissociated TCS and MTCS were found relatively stable to photodegradation (63). Furthermore, TCS and TCC cannot be removed by any sludge treatment method due to their hydrophobic nature. Therefore, their persistence and bioaccumulation in the soil are uncertain, leading to soil ecotoxicity, and carries a risk to enter food chains from biosolids-amended soil (64, 65). Widespread use of bisphenols and their analogs are responsible for their persistence in the water system. Bisphenol A found in the concentration of 31.51 ng/L and 332.75 ng/L in the tap water of all districts of Shanghai and surface water of China, respectively (66, 67). In addition to Bisphenol A, analogs such as Bisphenol A, S, E, F, and AF were detected in Yangtze River, China, in ranges 5.19–77.2 ng/L, 161–613 ng/L, and 47.5–353 ng/g in a colloidal and soluble phase, and suspended particulate matter form, respectively (68). Bisphenol A is also detected in a range of 2-199 ng/g in India’s Hooghly River, which is the reservoir of domestic wastes from surrounding districts (61). The concentrations of Bisphenol A in fresh and marine surface waters and sediments of North America and Europe were found to remain unchanged over more than a decade. However, the concentration was below the regulatory limits and published chronic toxicity values (69). 

Unlike other chemical ingredients of PCPs, there is little information about the fate and distribution pattern of ethanolamines, glycol ethers, and similar chemicals from PCPs into various environmental compartments. Among such a class of chemicals, monoethanolamine was found to persist on soil for decades in higher concentrations due to its strong ability to bind to soil. Though miscible in water but less likely to migrate into groundwater because of preferable presence as bound cations in the soil. However, ammonia generated as a result of bacterial degradation of monoethanolamine enters the groundwater. Monoethanolamine is found in a concentration of 400 to 3000 mg/kg in the soil at the US’s northeast chemical facilities.

Moreover, a higher amount of ammonia was observed in soil than the groundwater, *i.e.*, 500-1400 mg/kg *vs. *80-120 mg/L (70). Glycol ethers and their acetate have been used as a solvent in a variety of consumer products. Cleaning agents were the primary source of glycol ethers in Germany (71). Due to their volatility, such chemicals usually accumulate and contaminate indoor air (72). Volatile methyl siloxanes show temporal emission patterns. Various volatile methyl siloxanes (VMSs) were identified in Oporto’s region’s beach sand, Portugal, in concentrations between 0.007 to 17.8 ng/g_DW,_ and higher levels of all VMSs were observed during summer more than the winter season. Among them, cyclic VMSs and octocrylene were found prevalent in higher concentrations (73). Similarly, the urban air of Boulder, Colorado, represented a diurnal emission profile of D5 similar to that of benzene. The emission rate of D5 was higher between 6 and 7 AM later, which exponential decline at a constant rate of 9.2 h (74). Nearly 96% of PCPs utilized in Portugal showed VMSs in concentrations between 0.003 to 1203 μg/g. Thus, a higher amount of siloxanes are expected to be released in air and sewage systems through toiletries, especially shower gel and shampoo. The mean emission per capita of siloxanes observed was 1817 μg/day and 1607 μg/day in sewage systems and air, respectively. Furthermore, D5 and D3 found to be the predominant siloxanes in effluents while, D5 and D6 in the air (5). 

Human is unintendedly exposed to the fragrance, which is an aesthetic content of PCPs. Nearly 60% of fragrances through the water sewage system enters the general environment. Most wastewater treatment methods could not remove the fragrance compound; therefore, it ends up in rivers and streams (27, 75). A study detected synthetic musks in mussels of Asia–Pacific coastal waters suggesting ubiquitous contamination and widespread distribution of fragrances (76). In the Indian Hooghly river, the musk ketone is found in a range of 2-26 ng/g (61). A survey on the US household commodities reported concentrations of AHTN ranged from <5 ng/g to 451 µg/g, and HHCB ranged from < 5 ng/g to over 4000 µg/g, thus significantly detected in influent, effluent wastewater and surface water. The level of AHTN in the sediments and surface waters of Suzhou Creek was observed in a range of 2 to 31 ng/g and 8-20 ng/L, respectively. Similarly, the level of HHCB found in the sediments and surface waters of Suzhou Creek in a range of 3 to 78 ng/g and 20-93 ng/L, respectively (28, 77). Musk compounds persist in the aquatic environment due to their slow breakdown and accumulation in the fatty tissues of aquatic wildlife (75). Another study reported the air *vs. *lake concentration of AHTN and HHCB in Milwaukee, North America, and the levels were 2.9 *vs. *0.49 ng/m3 and 4.6 *vs. *1.1 ng/m^3^, respectively. Comparative to atmospheric deposition of musks, which was <1%, the WWTPs discharge was observed as the primary source of musk’s distribution into the environmental compartment, *i.e*., 3470 kg/year (78). Also, synthetic musk compounds (SMCs) were reported in concentrations ranging from 0.15 to 16.72 µg/L in Korean surface waters (56). In the case of insect repellents, especially DEET was detected in the European surface waters. The concentration of Bayrepel in the Sava River of Balkans and DEET in the Evrotas River of Greece was 105 μg/L and 5 μg/L, respectively. Such ubiquitous contamination was due to increased population and effluent wastewaters. However, Italy’s Adige river showed relatively low concentration levels of insect repellents (79). DEET was also detected in Africa’s groundwater at a concentration level of 1.8 μg/L (80). 

Several studies reported a wide distribution of parabens in the aquatic environment. Incomplete removal of parabens by the WWTPs causes them to release into the environment through WWTPs discharge and becomes the primary source of environmental pollution (81). Parabens were detected in a range of 87-593 ng/g in India’s Hooghly river water. The contamination was due to densely populated adjoining districts and Kolkata city (61). Seasonal changes also influence the fate of the aquatic distribution of parabens. The concentration of MP in China’s Yangtze river water was 2.72 ng/L, while the relatively highest paraben metabolite concentration, *i.e.*, p-hydroxybenzoic acid, was detected, *i.e.*, 510 ng/L (82). Recreational outdoor human activities contaminate pools and ponds with the PCPs chemicals. MP was identified as the most prevalent compound in the average concentration of 0.85 μg/L in 35 outdoor swimming pools in Changsha City of China (83). Parabens contaminate indoor air as well. Indoor dust samples collected from different athletic and residential facilities of US reported concentrations of MP, PP, EP, BP, and BePB were 1920, 965, 195, 80, and 6 ng/g, respectively (84). Like parabens, phthalates are also highly prevalent in our environment due to the anthropogenic activities of densely populated regions. Phthalates are one of the common micropollutants in the groundwater of different parts of India. The concentration of phthalic acid esters and diethyl hydroxylamine was observed in a range of 2-422 ng/g in the Hooghly river. Domestic wastes from Kolkata and adjoining districts enter the Hooghly river and considered as the main source of pollution and environmental distribution (61, 85). The metabolic product of phthalate diesters *i.e* phthalate monoesters found significantly prevalent in the freshwater of Okavango Delta, Botswana suggesting involvement of biosphere in their metabolism (86). Phthalates are semivolatile in nature thus ubiquitously present in the air of densely populated areas. Comparatively to other forms of phthalates, DEHP due to its higher molecular weight leaches out of the products and contaminates indoor and outdoor air and thus found more prevalent (59, 87). However, the indoor phthalates concentration observed to be higher than the outdoor air. For example, the total PAEs detected at student’s dormitory, residence, office and outdoor in Beijing were 468 > 498>280>125 ng/m^3^, respectively (88). While, the average concentrations of PAEs in Delhi, India were found relatively stable over the whole year *i.e* 703.1 ng/m^3^ probably due to its dense population (89). Moreover, occupational exposure of such chemicals observed to be higher than the general residential air, *e.g.*, higher concentration levels of phthalates regarded in the indoor air of saloon of Taipei, Taiwan, due to higher usage of hair care products (90). Similarly, the indoor dust samples collected from Vietnam detected higher phthalate concentrations ranging from 3440 to 106,000 ng/g (91). Similarly, dust samples from Qatar and Kuwait’s homes reported DEHP as the most prevalent in a concentration of 395 μg/g and 1704 μg/g, respectively (92, 93). In addition to DEHP, DBP was also detected in the street dust samples from Xi’an City in Northwest China where the mean concentration level of six PAEs was found to be 40.48 mg/kg (94). Soil samples collected from various areas of Tianjin, China reported different PAEs concentrations of PAEs with higher concentrations in the People’s Park *i.e* 0.92 μg/g than the XiLiu Park *i.e* 0.07 μg/g (95). Like air, DEHP and DBP found to be most abundant in soil samples taken from different areas of Tianjin including wasteland, vegetable, orchard, and suburban farmland. The overall concentration of PAEs identified in a range from 0.05 to 10.4 μg/g (96). 

**Figure 1:**
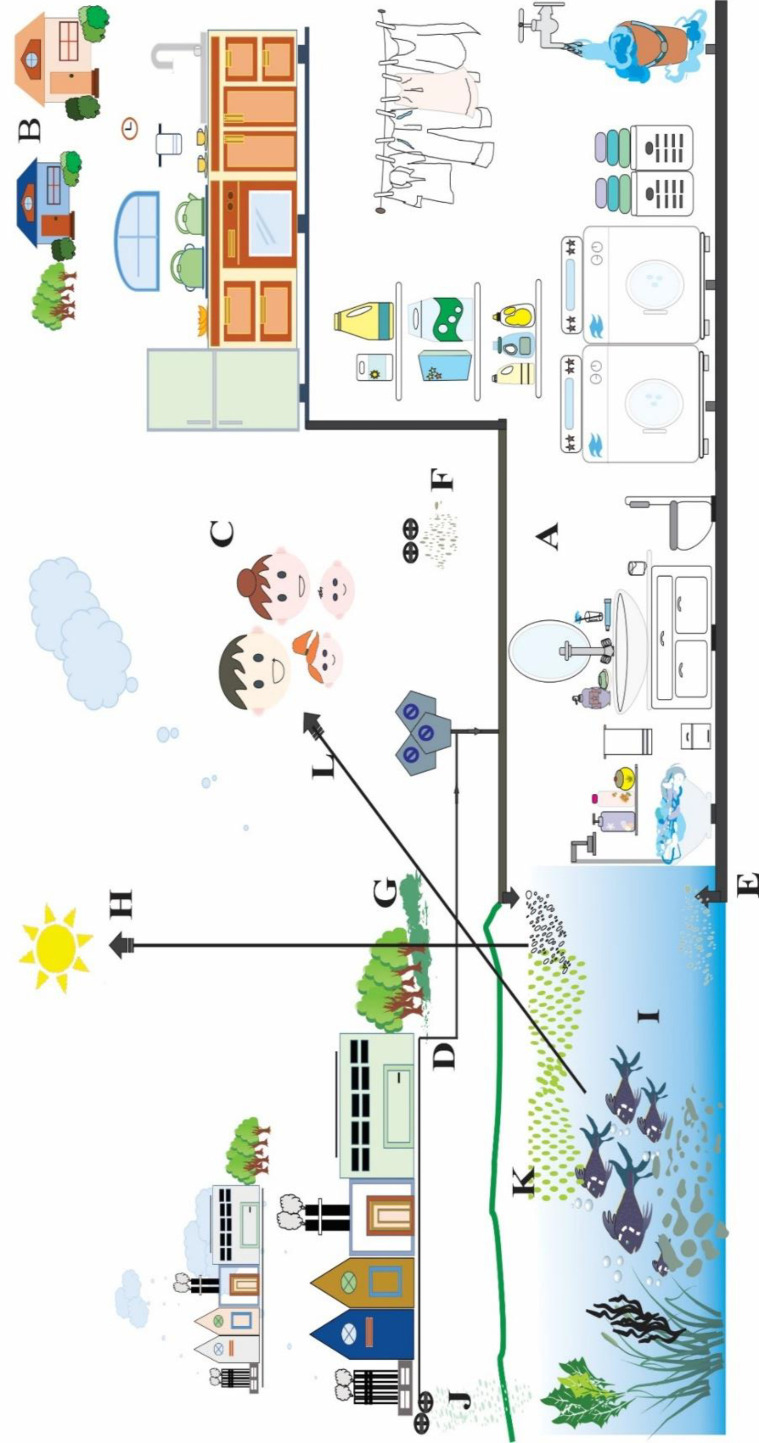
Distribution of emerging contaminants of PCPs in the environment. (A) PCPs contaminants through bathing, showering, cleaning, and washing activities; (B) Overpopulated areas increase the environmental burden of PCPs contaminants; (C) Outdoor activities and odor contaminate air; (D) Industrial and manufacturer wastes enter municipal wastewater; (E) Biological active and inactive components enter the aquatic water; (F) Landfill leaks contaminate groundwater; (G) PCPs contaminant sorb onto sludge and sediment that leads to contamination of agriculture land and soil; (H) Photodegradation of PCPs; (I) Contamination of aquatic wildlife; (J) Manufacturers PCPs wastes directly contaminate air, soil and water systems; (K) Polluted aquatic system facilitate algae overgrowth; (L) Contaminated aquatic wildlife enters the food chain

Environmental contamination of UV filters is heterogeneous from various sources of PCPs exposure. They either alone or other chemicals pollute the aquatic environment and wildlife (12, 39). Sewage leakages in urbanized areas and WWTPs effluent discharges were observed as the primary source of the spatial distribution of UV filters in Barcelona’s groundwater. Overall, UV filters were detected in a range from 20 to 55 ng/L in the groundwater, while BP4 was prominent in wastewater reservoirs and detected in a range of 738–1548 ng/L (97, 98). BP4 was also detected in Spain’s surface and drinking water in concentrations > 1 µg/L (99). Other prevalent UV filters identified in tap water from Catalonia were BP3, 2-ethylhexyl 4-methoxycinnamate (EHMC), 4-methyl benzylidene camphor (4MBC), OD-PABA, and OC determined in a range of 35-290 ng/L (100). A study on three different lakes of Japan reported sun-blocking agents ranging up to 4928 ng/L in surface water and from 2.0 to 3422 μg/kg_DW_ in sediments. Moreover, BS, BP3, EHMC, and OS were dominant in surface water since they receive wastewater effluents. While, HMS and OC were prevalent in sediments (101). In Germany, maximum concentrations of BP4 in raw wastewater reach up to 5.1 ug/L (102). The Glatt river water of Switzerland reported four UV filters to decrease concentrations BP4 > BP3 > 4MBC > EHMC. Their concentrations were identified using polar organic chemical integrative samplers and found BP4 in a range from 0.27 to 24.0 μg/POCIS while BP3, 4MBC, and EHMC were present in a concentration up to 0.1 μg/POCIS (103). In Portugal, the UV filters in beach sand from the Oporto’s region during the summer season reported their concentrations in a range from 0.030 to 373 ng/g_DW_ where 4MBC and BP3 were found more prevalent (73). The facts mentioned above confirm the widespread distribution and accumulation of various PCPs chemicals in the atmosphere, biosphere, and geosphere. Nevertheless, the primary sources are human encroachments, population, and activities thereby, polluting the environment directly through their uncontrolled consumption of PCPs. It is important to consider and monitor the potential of long term effects of various PCPs chemical exposure in the ecosystem for the health of humans and wildlife (Figure 1). 

Potential health effects

Most of the chemicals of PCPs act as EDCs, especially bisphenols, ethanolamines, TCS, phthalates or parabens (3, 36). EDCs are potentially carcinogenic, disturb metabolism, induce diabetes, obesity, and even infertility (104, 105). Exposure to mixtures of chemicals through the use of various kinds of PCPs at a time highlight the importance of considering combinations in health risk studies (59). This section explains some examples of adverse human health effects of emerging contaminants of PCPs from recently reported epidemiological studies. 

***Alkylphenol polyethoxylates***

APEOs and their metabolites act as endocrine disruptors. Food chain contamination with APEOs may put human health at risk. However, only a few epidemiological studies have evaluated APEOs effect on human health. Various PCPs ingredients such as bisphenol A (BPA), NP, and OP can interfere with human reproduction through multiple mechanisms. These chemicals can bind with sex hormone-binding globulin (SHBG) at high affinity, thereby disrupting natural hormones’ steroid-binding function (106). NP at different exposure levels can inhibit progesterone/androstenedione while induces testosterone/17β-estradiol production (107). Similarly, *in-vitro* studies showed an association between 4-tert-octylphenol (4tOP), 4-n-octylphenol (4OP), and 4NP with idiopathic male infertility and abnormal semen parameters (108). 4tOP exposure also decreases the human sperm viability and motility through *cAMP-PKA/PKC-*phosphorylation-mediated signaling pathway (109). Other possible reasons were OS-induced altered steroid hormones biosynthesis and hypothalamus-pituitary-adrenal axis activity (110). Similarly, another study found an association between urinary 4OP and impaired spermatogenesis (111). These facts confirm the endocrine-disrupting activity of phenolic compounds of PCPs and their risk of contributing to reproductive impairment. The US National Health and Nutrition Examination Survey (NHANES) during 2005-10 reported the association of urinary paraben and 4tOP with diarrhea and ulcerative colitis, respectively (112). Trillas and colleagues determined a modest association between occupational exposure of alkylphenolic compounds and breast cancer (113). Phenolic compounds also demonstrated unfavorable effects on pregnancy outcomes. *ABCG2* is a regulator of human placental ABC transporters that protects the fetus against various endogenous/xenobiotic combinations. Therefore, NP and BPA exposure found to downregulate placental *ABCG2* protein expression is consequently harmful to developing fetus (114). Another study reported NP’s presence in the maternal and umbilical cord plasma samples of pregnant women, *i.e*., found to be associated with higher 8-NO_2_Gua and 8-OHdG, suggesting OS or nitrative stress-induced possible risks of inflammation during pregnancy (115). A case of 55 year-old-woman reported with cholestatic hepatitis with fungicide ingestion. The fungicide composition included NP and methanol that were found responsible for irreversible hepatic injury and intracanalicular and intracytoplasmic cholestasis (116). 

*In-vitro* studies reported that phenolic estrogens such as NP could promote proliferation in different cells such as human uterine leiomyoma, lung adenocarcinoma, prostate non-tumorigenic epithelial and adenocarcinoma prostate cells through *TGF-β* signaling pathway, nuclear translocation of ERα, activation of ERβ, upregulation of EGFR and ERKs, increased gene expression of key regulators of inflammation and cell cycle along with increased IL-8 and IL-1β mRNA expression levels (117-120). Similarly, NP induces apoptosis in different human cell lines via the mitochondrial release of cytochrome C, caspase-3/9, and DNA fragmentation. Activation of A Disintegrin and Metalloprotease 17 along with various intracellular signaling pathways are other reported modes of NP induced apoptosis (121, 122). It was found to decrease the number of cells in G0/G1 phase while increasing the expression of ERK1/2 and phosphoinositide 3kinase p38, cFos, and SnoN thus affected colorectal cancer development through ERK and TGFβ signaling pathways (123). In addition to NP, NPEOs demonstrated toxicity to human keratinocyte and breast adenocarcinoma cell lines. It decreases cell viability via caspase-3, Poly (ADP-ribose) polymerase, and DNA dependent kinases activity, causing phosphorylation of histone H2AX that leads to DNA damage and double-strand breaks, therefore, become potentially genotoxic (124, 125). *In-vitro* studies suggest that environmental exposure to emerging contaminants from PCPs potentially adversely impacts human health. However, warranting further corroboration.

***Antimicrobials***

Antimicrobial contents in PCPs contribute to affect human health. The NHANES during 2003-04 identified the presence of TCS in urine samples, and its concentration was found to vary with the consumer age and socioeconomic status (126). Widespread use of TCS represents potential public health risk by developing antimicrobial resistance to *Escherichia coli* and *Salmonella enterica*. Development of co-resistance or cross-resistance might reduce the susceptibility of other clinically essential antimicrobials (127, 128). UVB or sunlight exposure of TCS at environmental intensities causes photodegradation and photoproducts formation of TCS through activation of *MAPK* pathway. Photosensitization induces the generation of ROS, free radicals (O2^•-^, ^•^OH), and lipid peroxidation via a type-I photochemical reaction. This resulted in endoplasmic reticulum stress, cell cycle arrest, lysosomal and mitochondrial destabilization in human skin keratinocytes cells. Other mechanisms were upregulated expression of caspase-3, cytochrome-C, *Bax*, phospho-p38, phospho-JNK while downregulated *Bcl-2* expressions (129). Long-term exposure to TCS exposure through PCPs is also deleterious for liver and kidney function. MTCS demonstrated toxic effects on human liver cancer cell lines through the caspase-dependent mitochondrial apoptosis pathway. Toxic effects were associated with increased ROS production, reduced *GSH/GSSG* ratio, and altered mRNA expressions with OS response, energy production, cell cycle regulation, and cell apoptosis. In addition, the reduced mitochondrial membrane potential and increased LDH release consequently resulted in decreased cell survival and cytotoxicity (130). 

Similarly, metabolomic and lipidomic analysis of normal L02 cells showed that TCS upregulated purine and amino acid metabolism, caused lipid accumulation, and disturbed energy metabolism along with ROS overproduction, altered antioxidant enzymes and lipid peroxidation resulting into TCS induced hepatotoxicity and hepatocarcinogenicity (131). β2-microglobulin is a biomarker for early kidney injury. The Korean National Environmental Health Survey during 2012-14 reported a positive association between urinary concentration of TCS and β2M, suggesting TCS exposure with the risk of developing kidney injury and CKD. However, it requires warranted more epidemiological studies to corroborate the facts as mentioned above (132). 

TCC acts as EDCs through its increased hormone-dependent induction of ER and AR-dependent gene expression and interferes with ER and the AhR regulon (133, 134). Epidemiological studies showed an association between antimicrobial exposure and poor reproductive health. TCS decreases sperm count and motility (135). OS might be the other reason indicated by the urinary levels of TCS and TCC and their association with 8-OHdG levels (136). In the female, TCS may contribute to the development of polycystic ovary syndrome (PCOS) (137). Moreover, TCC and TCS were found to disrupt human aromatase enzyme activity and decrease estradiol production in JEG-3 cells. Human aromatase produces estrogen from the androgen (AR) and maintains female reproductive function and pregnancy, while TCC and TCS are inhibitors of CYP19A1 (138). TCS induces eryptosis, which is characterized by erythrocyte hemolysis, shrinkage, and premature cell death. Cells lose membrane asymmetry and Ca^2+^ regulation through p38 *MAPK* and *RIP1* activation (139). TCS is also found to affect thyroid function, homeostasis, and autoimmunity. Higher urinary TCS concentrations were found inversely associated with free T_3_, thyroperoxidase, and thyroglobulin antibodies (140). 

The higher concentrations of TCS observed in plasma and mother’s milk using PCPs than in mothers who did not use TCS-containing products (22). Prenatal TCC and TCS exposure was found associated with the risk of adverse birth outcomes due to altered serum hormonal levels. Urinary TCC concentration of pregnant women found positively associated with serum levels of total T3 and T3/thyroxine (T4) ratio, while negatively related to thyroid-stimulating hormone (TSH) and SHBG. A positive association between urinary TCS concentration and serum estriol level suggests a potential risk to birth. Similarly, higher urinary concentrations of TCS and TCC in pregnant women were associated with a decreased gestational age.

In contrast, in TCS, the change in gestational age found infant sex-specific (141, 142). Moreover, exposure to TCS and some benzophenone found to change blood pressure during pregnancy in a fetal sex-specific manner (143). However, replicated studies are needed to strengthen these adverse facts of antimicrobial exposure on pregnancy through PCPs. A healthy placenta is vital for normal fetal development. Placental weight and placenta weight/birthweight ratio were found sensitive to the exposure of EDCs, including TCS, parabens, and phthalates (144). Another study reported TCS and TCC levels in maternal and umbilical cord blood samples from pregnant women diagnosed with fetal malformation, post-birth abnormalities, and decreased gestational age (142, 145 and 146). Prenatal and postnatal exposure to antimicrobials adversely contributes to the growth and development of children. Prenatal urinary concentration of TCS and MEP was found associated with early menarche in girls (147). Similarly, during gestation and later during childhood, urinary TCS was associated with more severe behavior problems in children. There observed increased externalizing issues, attention problems, hyperactivity, and somatization scores among children. The TCS-induced decreased circulating T4 levels and neuron apoptosis might be the reason for adverse effects on neurodevelopment (148). However, it requires further study to substantiate the results.

***Bisphenols***

The NHANES reported the presence of BPA in 93% of urine samples suggesting its widespread exposure (149). Epidemiological studies reported adverse reproductive health with BPs exposure. Urinary level of BPS in pregnant women was found associated with altered gestational age and lower birth weight of neonates, suggesting a deleterious effect on pregnancy and fetal growth (142, 150). However, prenatal BPA exposure was found to be positively related with birth weight (151). Furthermore, maternal BPA and BPS urinary concentrations were found related to a decreased gestational age and increased risk of preterm birth (152). BPs have anti-androgenic effects through altered TT levels. Urinary BPA concentration levels were found associated with TT levels in a sex-specific manner, with lower TT in adolescent boys than girls (153). Urinary BPA concentration was also related to the uterine leiomyoma incidence (154). This might be due to the capability of BPs to induce OS-mediated inflammation. Since higher urinary BPA concentration is associated with higher serum OHdG and isoprostane levels during pregnancy, it may result in OS-induced inflammation and DNA damage and future risk of adverse birth outcomes (155, 156). Urinary BPs concentrations of school children were measured, and it was found that children were commonly exposed to BPA, BPAF, BPF, and BPS. Among them, increased urinary BPA was found associated with increased 8-OHdG and 8-OHG levels suggesting the risk of oxidative DNA and RNA damage (157). The risk of diabetes was also found associated with BPA exposure (158). However, requiring additional research to rule out the exact mechanism. 

***Cyclosiloxanes***

Siloxane polymers may cause adverse effects via inhalation. It may induce pulmonary toxicity secondary to aspiration, especially in young children due to widely used PCPs (159). Cyclosiloxanes, especially octamethylcyclotetrasiloxane (D4) in low concentration, show ERα affinity and a weak estrogenic activity (160). Invitro exposure of D3, D4, and D5 in human breast epithelial cells demonstrated altered mRNA levels of DNA repair proteins such as ATM, ATR, BRCA1/A2, CHK1/K2. They were suggesting the potential of cVMS for inducing breast carcinogenesis through DNA damage and compromised DNA repair system (161). Epidemiological studies are required to evaluate the health risk associated with the use of cyclosiloxanes and related chemicals in PCPs. 

***Ethanolamines***

Ethanolamines reported skin allergies, irritation, and *Contact Dermatitis* Monoethanolamine has agonistic activity at histamine and muscarinic receptors, causing bronchoconstriction and asthma-like symptoms (162). Similarly, diethanolamine exposure induces asthma by a sensitization mechanism (163, 164). Monoethanolamine is an alkalizer used in hair color products. It induces scalp irritation and hair loss via reacting with hair protein. Oxidative hair damage is due to cysteine oxidation and cysteic acid generation, which leads to cuticle damage and protein loss (165). Diethanolamine has been frequently used in many skin products. Several cases reported skin allergy, atopic and contact dermatitis using products containing cocamide diethanolamine (166, 167). Some allergic contact dermatitis cases using triethanolamine-containing sunscreen had been reported (168, 169). However, epidemiological studies with larger sample sizes and various exposure levels are needed to confirm the health risks.

*Fragrances *

Fragrances are a mixture of VOCs that tend to break and mix with the dust or pollutants to form harmful secondary products or toxic air pollutants that are potentially more irritating or allergenic than the original substance (75). For example, terpenes from PCPs may react with indoor ozone to form secondary pollutants such as formaldehyde (170). Fragrances exacerbate symptoms of asthmatic patients and may induce atopic asthma. Fragrances with significant absorption in the UV range of 290-400 nm can cause phototoxicity and photoallergy resulting in dermal irritation and contact dermatitis (27). Dermatological patients often complain about hand eczema and allergy, mostly due to the ubiquitous usage of fragrances (27). Fragrance chemicals are also responsible for airborne contact and facial dermatitis (171, 172). The incidence of allergic contact dermatitis, hypersensitivity, and skin sensitization found to be higher among women than men due to their frequency of use of PCPs and ubiquitous presence of fragrance chemicals (173-175). Allergic contact dermatitis is also found to be common among children (176). Geraniol is a frequently used fragrance and potentially allergen in PCPs. It generates skin sensitizers such as geraniol-7-hydroperoxide, geranial, and neral upon autoxidation, responsible for contact allergy. Similarly, oxidized limonene and hydroperoxides of limonene also induce contact allergy and lymphomatoid contact dermatitis by using personal hygiene products (177, 178). Synthetic musk fragrances and their metabolites demonstrated estrogenic activity in human MCF-7 cells (179, 180). Polycyclic musks such as AHTN and HHCB interfere with estrogen receptors (181) AHTN and HHCB, AETT, AHMI also acts as ERβ, AR, and PR antagonists. AHMI and AHTN at low concentrations were found to have anti-progestogenic effects (181, 182). Similarly, talc powder’s perineal application is linked with human ovarian cancer (183, 184). 

Recently several safety assessment studies on fragrances ingredients have been performed. Fragrances ingredients were evaluated for respiratory/reproductive toxicity, genotoxicity, phototoxicity/photoallergenicity, skin sensitization, and environmental safety. Several fragrances such as methyl 2-nonenoate, Butyl 10-undecenoate, isobutyl propionate and 2-Isobutyl-4-methyltetrahydro-2H-pyran-4-ol, 4H-4a,9-Methanoazuleno[5,6-d]-1,3-dioxole, octahydro-2,2,5,8,8,9a-hexamethyl-, (4aR,5R,7aS,9R)-, (E,Z)-2,6-Nonadien-1-ol acetate, cyclohexyl acetate, p-Menth-8-en-2-one, Ethyl 2-hexylacetoacetate, 3-Methyldodecanonitrile, octyl crotonate, 4-ethyloctanoic acid, 1-cyclohexene-1-acetic acid, ethyl acetoacetate, isobutyl hexanoate, Isopropyl acetate, p-dimethoxybenzene, m-dimethoxybenzene, ethyl benzoate, ethyl 3-phenylpropionate, 3,7-dimethyl-6-octenoic acid, 3-(4-methylcyclohex-3-enyl)-3-butenyl acetate, 2-octanone, and 2-ethyl-5-methoxybicyclo[2.2.1]heptane, butyric acid *etc.* were found not to be the ‘Persistent, Bioaccumulative and Toxic’ and were found safe as per the International Fragrance Association Environmental Standards (185-208). 

***Glycol ether***

Urinary concentration of glycol ether metabolites such as butoxyacetic acids, ethoxyacetic acid, and phenoxyacetic acid was associated with a long time to pregnancy. Among other metabolites, phenoxy acetic acid is the primary metabolite of 2-phenoxyethanol, significantly associated with a longer pregnancy time. It thus might be a plausible risk for decreased fecundability in the female of reproductive age (209). In men, glycol ether was found to reduce motile sperm count (210). Glycol ether exposure was found lethal for developing children. A study reported glycol ether metabolites, *i.e*, phenoxy acetic acid and ethoxy acetic acid, in maternal urine samples. Prenatal urinary concentration of these metabolites was found associated with the lower Wechsler Intelligence Scale for Children IV verbal comprehension index scores and the Developmental Neuropsychological Assessment scores among children of 6 years, suggesting a deleterious impact on the neurodevelopment and neurocognitive performance of infants and children (211). Glycol ether induces multiple allergic symptoms, including asthma, eczema, rhinitis, and IgE sensitization (37). Another study reported skin reactions, irritation, and skin erythema among healthy individuals to toothpaste contents, including propylene glycol and sodium lauryl sulfate (212). Similarly, a case of allergic contact dermatitis to ethylhexylglycerin, and pentylene glycol was reported in a woman (213).

***Insect repellents***

There are sparse data on the safety of insect repellent exposure in humans. Unintentional ingestion of insect repellent was found to be associated with easily manageable minor toxicities. The minor adverse symptoms related to insect repellent exposure included oral/ocular irritation/pain, red eye, conjunctivitis, and vomiting that could be managed outside the healthcare facility (214). However, a single case of 39 years older man reported erythematous-edematous dermatitis characterized by widespread, persistent itching on limbs was due to picaridin content of insect repellent aerosol (215). Another case of 22 years old male reported contact urticaria upon application of DEET containing repellent (216). However, an *in-vitro* study on endothelial cells demonstrated that DEET exposure stimulates angiogenesis, cellular proliferation, migration, and adhesion, leading to the risk of tumor growth and the carcinogenic potential of DEET (217). However, further studies with a large human sample at different exposure levels are warranted. 

***Parabens***

A survey conducted in the US reported that 60% of PCPs contain at least one paraben (218). Even at minimum human exposure level, paraben could accumulate in the body tissues, *e.g.*, MP accumulates in human breast tissues (219). There was observed MP and PP in >96% of human urine samples (220). However, more research is required to find the exact body burdens of these chemicals. Parabens from PCPs are responsible for any adverse health effects. Paraben exposure is potentially harmful to the reproductive system (221). Parabens, including MP, BP, and EP, were associated with decreased sperm count and motility. Hydroxylated paraben metabolites adversely affect male reproductive health (135). The concentrations of parabens used in commercially available PCPs may impair sperm viability via the generation of mitochondrial ROS and oxidative DNA damage (222). Parabens were found to intact human breast tissues and identified in considerable concentrations in breast tumors (219). A study reported urinary MP and PP concentrations and their association with the high risk of breast cancer (223). However, warrant further research to prove this association. Prenatal urinary concentration of BP, MP, and PP found associated with decrease serum concentration of SHBG and PP with lower maternal TSH, thereby suggesting the potential risk of these endocrine disruptors to birth outcomes (141, 224). In pregnant women, MP and PP’s urinary concentration was found associated with increased gestational age (141). Similarly, urinary and umbilical cord blood plasma BP concentrations were associated with decreased gestational age and birth weight while PP with decreased neonates’ body length (225). Among children, urinary paraben concentration was found associated with early menarche. Urinary concentration of MP was found associated with early breast and pubic hair development in girls, while early genital development in boys with urinary PP. Such changes were common among children more likely to use PCPs (147). Prenatal paraben exposure was also found to be associated with impaired child cognitive abilities. For example, prenatal MP concentration was found associated with lower mental development index scores among girls (226). However, further human and animal studies are needed to elucidate the facts mentioned above’ biological mechanism.

***Phthalates***

A Survey conducted in the US reported that 60% of PCPs contain at least one phthalate (218). Phthalates metabolize quickly, and due to short half live do not accumulate in the body and primarily excreted through the urine (227). Urine samples of US children in a TESIE study reported 13 of 19 phthalates or non-phthalate replacement metabolites (8). Phthalates in the dust cause contact allergy, *e.g.*, butyl benzyl phthalate irritates skin, rhinitis, and eczema. Similarly, di(2-ethylhexyl) phthalate irritates the respiratory tract and causes asthma in children (170). Prevalence of asthma and allergy among US children was found associated with monocarboxyisooctyl phthalate, which was found responsible for lower forced expiratory volume and poorer respiratory health (228). Another study reported an association of prenatal urinary concentration of phthalate with the altered blood level of T helper 1 and 2 cells in their children. Mono-n-butyl phthalate and methylparaben were associated with higher T helper 2 and lower T helper 1 cell levels.

Moreover, children presented a significant association of atopic cytokine profile with poor lung function. Therefore, suggesting the potential risk of decreased lung function, asthma, eczema, and aeroallergens among children with increasing age. (229). There are globally growing concerns related to heart and kidney diseases with the exposure of PCPs contaminants. Recently a study showed a strong association between urinary concentration of monobutyl phthalate and albumin-to-creatinine ratio. The albumin-to-creatinine ratio is the kidney function marker and is found sensitive to phthalate exposure suggesting its potential risk of developing CKD (230).

Nevertheless, urinary phthalate was associated with altered liver function, cardiometabolic risk factors, insulin resistance, visceral adiposity index, and lipid accumulation product. Urinary monoethyl phthalate concentration was associated with serum transaminase levels, triglyceride, visceral adiposity index, lipid accumulation product, and high-density lipoprotein cholesterol levels. Similarly, Urinary mono-(2-ethylhexyl) phthalate was found associated with increased body mass index. They were suggesting the potential risk of liver and heart disease through phthalates (231). Phthalates act as EDCs. Urinary diethyl hexyl phthalate metabolites in adult men decrease steroid hormone levels (232, 233). Likewise, the urinary metabolites of DBP, BBP, DEP, and DINP demonstrated anti-androgenic activity (234). That’s why prenatal exposure of phthalate was found to adversely affect the male reproductive system (235).

Similarly, the urinary concentration of phthalates monoester and oxidative metabolites can alter semen quality at their general population exposure levels (236). For example, among children, phthalates metabolites of low molecular weight are found associated with behavioral problems, aggression, attention problems, conduct problems, and depression (237). Phthalates exposure was also found associated with early menarche. Prenatal urinary monoethyl phthalate concentrations were found associated with the early onset of pubic hair development in girls (147). Similarly, plasma phthalate levels were found associated with an increased prevalence of precocious puberty. The most prevalent diethylhexyl phthalate metabolite levels were found significantly associated with precocious puberty, especially among girls who are more likely to use different PCPs and cosmetics (238). In males, there was a significant association between maternal urinary concentration of phthalate and utero penile growth and development in different races. African Americans showed a significant influence of maternal phthalates on penile development and measurements (239).

***UV filters***

A wide range of UV filters in PCPs act as EDCs (240). The NHAHES during 2003-04 reported the presence of benzophenone-3 in 96.8% of urine samples suggesting its ubiquitous exposure (241). Therefore, UV filters absorb harmful UVB radiations, which are added as an essential ingredient of PCPs and plastic products to protect them against UV rays-induced deterioration. Octyl salicylate is a water-insoluble and weak UV absorber though not free from skin sensitization. Few cases of allergic contact dermatitis in older women were reported using sunscreen creams, *e.g.*, Olay Total Effects™. The symptoms of skin irritation, tightness, and redness were associated with the creams’ Octyl salicylate ingredient (242, 243). Moreover, few allergic contact dermatitis cases were reported using lip care balm containing polysilicon-15 as a UV filter (244). Similarly, phenyl salicylate content of PCPs and plastic products such as galenic creams, lip salve, and industrial safety spectacles were found associated with the cases of contact dermatitis and eczema (245, 246). UV filter was associated with various endocrine disorders among females of reproductive age, such as PCOS, uterine leiomyoma, and endometriosis. There observed a positive association between the risk of PCOS and urinary concentration of octocrylene. The risk was increased with increased body weight and body mass index > 24 (247). Similarly, benzophenone-type UV filters were found associated with the incidence of uterine leiomyoma and endometriosis. Urinary concentrations of 2,4-dihydroxy benzophenone and 2-hydroxy-4-methoxybenzophenone were found related to the risk of fibroids and endometriosis diagnosis (154, 248). Among men, the urinary concentration of UV filters was found to alter semen quality. For example, 2,2’,4,4’-tetrahydroxybenzophenone decreases sperm concentrations, limits sperms mobility and maturity. Similarly, 2,2’-dihydroxy-4-methoxybenzophenone is associated with the higher acrosomal area and decreased hypo-osmotic swelling (249). The aforementioned mentioned facts suggest that exposure to UV filter has negative implications on reproductive health. For example, urinary concentration of 2,2’,4,4’-tetrahydroxybenzophenone in male partners found associated with reduced fecundity that resulted in a longer time to pregnancy (250). These chemicals are also harmful to neonatal development; for example, Hirschsprung’s disease was found associated with prenatal benzophenone-3 exposure. Hirschsprung’s disease is the intestinal abnormality in neonates characterized by the failure of enteric neural crest cell migration during embryogenesis. Maternal urinary concentration of benzophenone-3 was found to induce cytotoxicity and expression of receptor tyrosine kinase. BP3 suppressed cell migration via *SLIT2/ROBO1*-miR-218-*RET/PLAG1* pathway. Furthermore, it regulates receptor tyrosine kinase expression, miR-218, *PLAG1, SLIT2*, and *ROBO1*, thereby found to be associated with Hirschsprung’s disease (251) (Table 2).

**Recommendation and perspectives**

Several strategies could be adopted to protect the environment and living beings from the harmful exposure of emerging contaminants of PCPs. Physical-chemical properties and product concentration of these contaminants predict their fate of distribution and the mode for their removal from the environment. For example, the biological treatment process was more effective for removing synthetic musk compounds than chemical treatment, filtration, or disinfection processes (56). Similarly, an advanced water treatment process based on granular activated carbon filtration demonstrated improved efficiency for removing PCPs contaminants compared to conventional drinking water treatment plants (253, 254). Therefore, this system is rapid and compact, especially for water treatment involving combined sewer overflows (255). Similarly, the addition of pelletized fine-grained activated carbon was observed as an established method for remediation. Comparative to granular activated carbon, the addition of pelletized fine-grained activated carbon on contaminated sediments of Lake Apopka for 30 days demonstrated superior performance for mitigating the organochlorines uptake by the aquatic organisms as shown by the limited *Lumbriculus variegatus* (blackworm) bioaccumulation (256). Recently, algae-based systems consisting of *Scenedesmus obliquus* and *Chlorella vulgaris* suggested the transfer and transformation mechanism of PCPs removal. For example, in addition to phototransformation and photolysis of TCS, the synergistic relationship between the algae and bacteria facilitate its biotransformation. This suggests that algae in lagoons and open-water surface-flow wetlands can effectively contribute to algae-based passive treatment systems (257). Similarly, *Nannochloris* sp based algae-mediated sorption contributed to 27% of the removal of TCS from the US Lake Mead water. However, algae-mediated contaminants uptake may potentially affect the food web and contribute to aquatic species toxicity (258).

Despite strict rules and regulations of governing bodies, loopholes in the existing system facilitate the ubiquitous presence of PCPs. There is a need to establish clear policies for PCPs sale and distribution as either over the counter, drug, or cosmetics. All intended ingredients, their concentrations, and instructions for frequency of use as per age groups must be clearly labeled. They must be marketed only after passing cumulative toxicological analysis, safety, and efficacy studies. Harmony among rules and regulations of different countries will also limit their redundant distribution. Several PCPs demonstrated the ‘off-label’ use of chemicals. For example, repellents such as Lilial and methyl dihydro jasmonate were used in the Ivanka Trump eau de parfum and Bombshell® fragrances. These perfumes may repel mosquitoes, but their ‘off-label’ use is not recommended. Therefore, it is essential to mention all intentional ingredients of PCPs on their label (259).

Similarly, the concentrations of antimicrobial must also be listed. Disclosure of product ingredients would also enable researchers to identify exposures for study and risk evaluation and allow consumers to make decisions consistent with their values. Eco-labelling is a market-driven and product-specific approach introduced for one major group of PCPs, including shampoo, shower gels, and foam baths. The purpose is to reduce the discharge of environmentally harmful components of such products. It may help public awareness and promote ecologically compatible products (260, 261), and a similar system of labeling may be developed for a different chemical class of PCPs. Today’s man is exposed to chemicals from multiple product types. Toxicity testing and chemical risk assessment generally do not address the effects of combined exposures; therefore, the potential of combined risks often remained underestimated. A study reported cumulative risk assessment of multiple chemicals via various routes and pathways and found the incremental risks and exposure levels exceed the acceptable human levels for many chemicals. Therefore, it is vital to consider the cumulative toxicological effects of combined exposures (262).

Similarly, a ratio of acceptable to consumer exposure levels for a chemical more than 1 means a product free from skin sensitizing potential. For example, a perfume reported a fair to consumer exposure levels ratio values for citral, HICC, isoeugenol, methyl 2-octynoate, and Lilial below 1, suggesting potential for skin sensitization and found not safe. Therefore, the manufacturers must reduce PCPs chemicals’ content according to the appropriate human safety level (263). 

Several cosmetic companies introduced patch test kits for the consumer to detect spot content sensitivities. A UK-based company introduced a patch test kit for hairdressers. Only three out of seven p-phenylenediamine allergic patients were identified using this system, suggesting low reliability and sensitivity of such home diagnostic patch test kits for consumer protection. This also highlights serious concerns about their distribution and regulations (264). However, another study reported successful results of a commercially available formaldehyde spot test kit. Nine out of 10 formaldehyde spot test kits successfully identified the presence of formaldehyde in PCPs.

Moreover, compared to enzymatic-based tests, the chemical-based formaldehyde spot test was more reliable for formaldehyde content identification (265). Patch testing is standard for the identification of allergic *Contact Dermatitis* Commercially available kits usually do not carry all cosmetic-specific antigens and therefore lack sensitivity and specificity. Consequently, it is suggested to develop and introduce patch test kits with improved sensitivity and specificity for various antigens found in PCPs (173). However, upon accidental ingestion, consumers are advised to contact poison control/information centers (30). There reported a case of bradycardia in a child upon accidental ingestion of brimonidine as toothpaste (266). Nearly 49% of acute fluoride toxicity and lethality incidence were reported in New Jersey from accidental ingestion of fluoride toothpaste among children between 18 months to 3 years. However, all adverse events were home treated in time with calcium antidote (267). Therefore, consumers are encouraged to contact such centers in case of need of any help or guidance. According to the Freedom of Information Act request, several PCPs and cosmetics have been recalled from 2002 to 2016. The most common among them was the baby products. Other reasons were products with bacterial contamination, labeling issues, unapproved component, and skin irritation. Therefore, a dermatologist reporting adverse events could strengthen public safety and encourage recalls of harmful products, similarly, consumer’s responses could bring meaningful improvement in existing PCPs related regulations (268). 

Green chemistry research is aimed at identifying and developing functional alternatives that do not have endocrine-disrupting activity. For example, alkenones is a non-animal derived wax from marine microalgae that offers a sustainable non-petroleum-based structuring agent for lipsticks. Similarly, biosurfactant obtained from *Lactobacillus paracasei* provides an attractive option of non-petroleum based surfactant for the formulation of chemical-free formulations, *e.g.*, toothpaste, creams, and shampoo, *etc.* suggesting more research to find an environment-friendly and better alternative to chemical ingredients of PCPs (269, 270). To achieve the tasks mentioned above, there is a need for collaboration between different institutes involving manufacturers, industries, regulatory authorities, research centers, and medical institutes to establish a system for effective reporting and evaluation of adverse events related to the use of PCPs. There is a need to identify and address harmful and/or problematic content of PCPs and find suitable alternatives. Manufacturers need to perform safety testing through all exposure routes and address them honestly on the product’s label (75). Finally, raise consumer awareness and information regarding product safety and contents efficacy regarding their frequent use (3). Motivating and educating them to read the product label, investigating the safety of a product through web-based databases, and rationalize PCPs selection based on EDCs –free content. These strategies could help in a measurable reduction of EDCs exposure of 27-45% (271). Moreover, public places and institutions such as colleges, universities, and hospitals can be made free from the exposure of EDCs by use of PCPs, *e.g.*, soap dispensers and toiletries based on EDCs free, natural, and safe ingredients (3). Based on the evidence-based risks associated with emerging contaminants of PCPs to human, wild and marine life and the environment, it is highly desirable to shift from chemical-based to chemical-free products and highly recommended for simple life-style (Table 3). 

**Table 1:** Sources of environmental contaminants in personal care products

**Environmental contaminants in PCPs**	**Source of exposure**	**References**
Alkylphenol polyethoxylates (APEOs): nonylphenol (NP), nonylphenol polyethoxylates (NPEOs), octylphenol (OP), 4-n-octylphenol (4OP), 4-tert-octylphenol (4tOP), octylphenol ethoxylates (OPEs)	Detergents, disinfectants, surface cleaners	(7, 35)
Antimicrobials: 1,4-dichlorobenzene, *ortho*-phenylphenol, triclocarban (TCC), triclosan (TCS)	Deodorants, detergents, toothpastes, soaps,	(7, 22, 36)
Bisphenols: bisphenol A (BPA), bisphenol B (BPB), bisphenol F (BPF), bisphenol AF (BPAF), bisphenol S (BPS),	Conditioners, detergents, lotions, nail polishes, shampoos, shaving creams, soaps, sunscreens	(7, 23)
Cyclosiloxanes: hexamethylcyclotrisiloxane (D3), octamethylcyclotetrasiloxane (D4), decamethylcyclopentasiloxane (D5), dodecamethylcyclohexasiloxane (D6)	Antiperspirants, baby lotions, baby oils, baby shampoos, deodorants, diaper creams, fragrances, hair care products, lotions, nail polishes, skin cleansers	(25)
Ethanolamines: monoethanolamine (MEA), diethanolamine (DEA), triethanolamine (TEA)	Cleaners, detergents, shampoos	(7)
Fragrances: cashmeran (DPMI), celestolide (ABDI), galaxolide (HHCB), phantolide (AHDI), toxalide (AHTN), traseolide (ATII)	Air fresheners, body creams, cleaners, deodorants, detergents, fabric softeners, facial cleanser, lotion, soaps, sunscreen	(7, 27, 28)
Glycol ethers: 2-methoxyethanol (ME), 2-ethoxyethanol (EE), 2-isopropoxyethanol (IPE), 2-butoxyethanol (BE)	Cleaners, face lotion, polish/wax, sunscreen, and shaving cream	(7, 37)
Insect repellents: bayrepel (BR), ethyl butyl-acetylamino propionate (IR3535), indole (ID), N,N-diethyl-m-toluamide (DEET), picaridin, piperonyl butoxide (PBO)	Creams, lotion, spray containing insect repellents	(29, 30)
Parabens: methylparaben (MP), ethylparaben (EP), propylparaben (PP), butylparaben (BP), benzylparaben (BePB)	Blush, cosmetics, foundation, mascara, sunscreen	(7, 38)
Phthalate: benzyl butyl phthalate (BBP), di (2-ethylhexyl) phthalate (DEHP), di (2-ethylhexyl) terephthalate (DEHTP), dimethyl phthalate (DMP), di-isobutyl phthalate (DiBP), di-n-butyl phthalate (DBP), di-isononyl phthalate (DiNP)	Antiperspirants, cosmetics, deodorants, diaper creams, body creams, body lotions, baby oils, fragrances, hair gels, hair sprays, mousses, nail polishes, skin cleansers, shampoos	(8, 33)
UV filters: benzophenone-1 (BP1), benzophenone-2 (BP2), benzophenone-3 (BP3), benzophenone-4 (BP4), 3-benzylidene camphor (3BC), benzyl salicylate (BS), 4,4’-dihydroxybenzophenone (4DHB), 2-ethylhexyl 4-methoxycinnamate (EHMC), ethoxylated ethyl 4-amino benzoate (PEG25-PABA), ethyl-4-aminobenzoate (Et-PABA), homosalate (HMS), 4-hydroxy benzophenone (4HB), isopentyl-4-methoxycinnamate (IMC), 4-methylbenzylidene camphor (4MBC), octocrylene (OC), octyl dimethyl para amino benzoate (OD-PABA), octyl-methoxycinnamate (OMC), octyl salicylate (OS), para amino-benzoic acid (PABA), phenyl salicylate (PS) titanium dioxide (TiO_2_) zinc oxide (ZnO)	Face creams, skin lotion, sunscreen lotion	(34, 36, 39-41)

**Table 2:** Potential health risks associated with emerging contaminants of PCPs

	**Study**	**PCPs contaminants**	**Potential health risks**	**References**
	**Alkylphenol polyethoxylates**				
35	Human	Fragrances	Skin sensitization, contact hypersensitivity, Allergic contact dermatitis	(174, 175) (176)
36	Human	Fragrances	Facial dermatitis	(171)
37	Human	Fragrances	Airborne contact dermatitis	(172)
38	Human	Geraniol	Contact allergy	(252)
39	Human	Limonene	Contact allergy	(177)
40	Human	Limonene	Lymphomatoid contact dermatitis	(178)
	**Glycol ether**				
41	Human	Glycol ether metabolites	Negative impact on the neurodevelopment of infants and children	(211)
42	Human	Glycol ether	Motile sperm count	(210)
43	Human	Glycol ether metabolites	Decreased fecundability & longer time to pregnancy	(209)
44	Human	Pentylene glycol	Allergic contact dermatitis	(213)
45	Human	Propylene glycol	Skin reaction, irritation, skin erythema	(212)
46	Human	Glycol ether	Allergic symptoms, asthma, eczema, rhinitis & IgE sensitization	(37)
	**Insect repellents**				
47	Human	Insect repellents	Oral irritation/pain, red eye, conjunctivitis & vomiting	(214)
48	Human	N,N-diethyl-m-toluamide	Contact urticaria	(216)
49	Human	Picaridin	Erythematous-oedematous dermatitis	(215)
	**Parabens**				
51	Human	Butylparaben & propylparaben	Adverse birth outcomes decreased gestational age birth weight & body length of neonates	(225)
52	Human	Methylparaben & propylparaben	Increases gestational age	(142)
53	Human	Methylparaben, butylparaben & propylparaben	Endocrine disruptor decreased serum sex hormone-binding globulin & the potential risk to birth outcomes	(141)
54	Human	Methylparaben & propylparaben	Breast cancer	(223)
55	Human	Methylparaben & propylparaben	Early menarche, breast & pubic hair development in girls & genital development in boys	(147)
56	Human	Methylparaben, butylparaben & ethylparaben	Decreases sperm count & sperm motility	(135)
57	Human	Methylparaben	Impairs child cognitive abilities	(226)
58	Human	Propylparaben	Lowers maternal thyroid-stimulating hormone during pregnancy	(224)
1	Human	4-tert-octylphenol	Ulcerative colitis	(112)
2	Human	4-n-octylphenol	Impairs spermatogenesis	(111)
3	Human	Alkylphenolic compounds	Breast cancer	(113)
4	Human	Nonylphenol	Cholestatic hepatitis	(116)
5	Human	Nonylphenol, 4-tert-octylphenol & 4-n-octylphenol	Idiopathic male infertility	(108)
6	Human	Nonylphenol	Increase oxidative & nitrative stress	(115)
7	Human	Nonylphenol & bisphenol A	Adverse pregnancy outcomes	(114)
8	Human	4-tert-octylphenol	Reduce fetal penis length	(110)
	**Antimicrobials**				
9	Human	Triclosan	Bacterial resistance	(127, 128)
10	Human	Triclosan	Eryptosis/erythrocyte hemolysis	(139)
11	Human	Triclocarban, triclosan & butylparaben	Decreases/alters gestational age at birth	(142, 146)
12	Human	Triclocarban, triclosan	Adverse birth outcomes	(141)
13	Human	Triclosan, parabens & phthalates	Alters placental weight & placental–to–birth weight ratio	(144)
14	Human	Triclosan	Fetal malformations	(145)
15	Human	Triclosan	Behavior problems in children, hyperactivity & attention problem	(148)
16	Human	Triclosan & benzophenones	Blood pressure changes in fetal sex specific manner	(143)
17	Human	Triclocarban & triclosan	Oxidative stress	(136)
18	Human	Triclosan & monoethyl phthalate	Early menarche in girls	(147)
19	Human	Triclosan	Affects thyroid homeostasis & autoimmunity	(140)
20	Human	Triclosan	Polycystic ovary syndrome	(137)
21	Human	Triclocarban & triclosan	Negatively affect female reproductive function	(138)
22	Human	Triclosan	Kidney injury & chronic kidney disease	(132)
	**Bisphenols**				
23	Human	Bisphenol S	Small & long gestational age	(142)
24	Human	Bisphenol A	Alters birth weight	(151)
25	Human	Bisphenol A & bisphenol S	Decrease gestational age & risk of preterm birth	(152)
26	Human	Bisphenol S	Lower birth weight of female child	(150)
27	Human	Bisphenol A	Anti-androgenic effects	(153)
28	Human	Bisphenol A	Uterine leiomyoma	(154)
29	Human	Bisphenol A	Oxidative DNA damage	(156)
30	Human	Bisphenol A	Oxidative stress	(155)
31	Human	Bisphenol A, S, F & AF	Oxidative DNA & RNA damage	(157)
	**Ethanolamine**				
32	Human	Diethanolamine & monoethanolamine	Atopic contact dermatitis	(166, 167)
33	Human	Triethanolamine	Atopic contact dermatitis	(168, 169)
34	Human	Monoethanolamine	Hair loss & scalp irritation	(165)

**Table 3:** Recommendation for reducing environmental exposure of emerging contaminants of PCPs

Granular activated carbon filtration has improved efficiency for the removal of PCPs contaminants from water Pelletized fine-grained activated carbon is a superior method for remediation The biological treatment process is more useful for the removal of synthetic musk compounds Algae-based systems involving the synergistic relationship between algae and bacteria facilitate biotransformation and PCPs removal Harmony among PCPS related rules and regulations regarding different countries must be considered All intended ingredients, their concentrations, and instructions for frequency of use as per age groups must be clearly labeled on PCPs ‘Off-label’ use of chemicals must be prohibited Eco-labelling may be considered for various chemical groups of PCPs Cumulative toxicological effects of combined exposures must be analyzed before product marketing Reduce the content of PCPs chemicals according to the appropriate human safety level Highly specific and valid home diagnostic patch test kits for consumer protection may be introduced Contact poison control/information centers upon accidental ingestion of PCPs chemicals Consumers must inform PCPs related adverse events and participate in the product recall mechanism Encourage green chemistry research aimed at identifying and developing functional alternatives to EDCs Encourage collaboration between different institutes for research and human health purposes Raise consumer awareness and information regarding product safety and contents efficacy Public places and institutions may be provided with EDCs free PCPs *e.g.*, soap dispensers and toiletries

## Conclusion

Evidence-based facts have confirmed the risks of serious adverse effects associated with various chemical contaminants of PCPs, which are not limited to an individual but spread to the entire ecosystem involving human, wild, and marine life. The increased availability and diversity of PCPs are responsible for the higher loading of emerging contaminants of PCPs and their biologically active and inactive components that have been persistently and continuously releasing in the atmosphere, biosphere, and geosphere. It is now essential for all regulatory authorities of different countries to develop standard rules and policies for regulating various aspects of PCPs manufacturing, sale, and distribution. There is also a need to harmonize regulations of different countries so that a joint check and control system may be employed across the borders to ensure consumers’ safety and efficacy and preserve environmental pristine. Considering consumer’s rights, all intended ingredients, their concentrations, and instructions for frequency of use as per age groups may be clearly labeled on packages of PCPs. Public awareness and education regarding the PCPs content, application, and service could further rationalize the consumer’s usage patterns. Moreover, collaboration among different research institutes to encourage the development of chemical-free and natural products may offer a consumer safe and better alternatives to chemical-based PCPs. In conclusion, the emerging environmental contaminants of PCPs and their association with the growing risks of negative effects on human health and globally on the environment emphasize the chemical-free simple lifestyle. 
